# Earthworm Species from Diverse Ecological Groups Negatively Affect Enchytraeid Density in a Forest Ecosystem

**DOI:** 10.3390/biology14091283

**Published:** 2025-09-17

**Authors:** Kamil Karaban, Anita Kaliszewicz, Krassimira Ilieva-Makulec, Alexei V. Uvarov

**Affiliations:** 1 Institute of Biological Sciences, Cardinal Stefan Wyszynski University in Warsaw, Wóycickiego 1/3, 01-938 Warsaw, Poland; a.kaliszewicz@uksw.edu.pl (A.K.); k.makulec@uksw.edu.pl (K.I.-M.); 2 Institute of Ecology and Evolution, Russian Academy of Sciences, 119071 Moscow, Russia; av.uvarov@hotmail.com

**Keywords:** soil ecology, density gradient, earthworms, enchytraeids

## Abstract

Earthworms and enchytraeids are two very important groups of soil organisms that influence soil biology and ecology, as well as physicochemical processes occurring in the soil. The interactions within and between these major groups of soil fauna are currently among the most pressing topics in soil ecology and are still insufficiently understood. In a field mesocosm experiment, we examined the effects of the density of the following five key earthworm species in Central Europe: *Dendrobaena octaedra*, *Lumbricus rubellus*, *L. terrestris*, *Aporrectodea caliginosa*, and *Allobophora chlorotica*. These species were selected to represent the following three major ecological groups of lumbricids: epigeic, endogeic, and anecic. The mesocosm experiment examined the effect of these species across density gradients and at two soil profile horizons—litter and mineral soil—and at the entire soil profile within the mesocosm. This comprehensive and unique approach was used to compare the effects of earthworm density gradient on enchytraeid abundance under identical conditions in the forest soil. The results indicate that all studied species of earthworm negatively affected enchytraeid density. The strength of this effect depended on the earthworm species, earthworm density, and the level of the analyzed soil profile. Epigeic *L. rubellus,* endogeic *Aporrectodea caliginosa*, and anecic *L. terrestris* appeared to be the most effective. Higher earthworm density had a greater effect on the density of enchytraeids. The most significant results concerned the litter layer, where the density of enchytraeids was highest due to their natural occurrence. Our results provide new insights into the ecological relationships among key groups of soil fauna and can be a starting point for predicting changes caused by earthworms in newly occupied ecosystems.

## Simple Summary

Earthworms and enchytraeids are known as soil engineers, modifying soil structure, microorganisms’ activity, and altering nutrient availability for other organisms. Through their activities, earthworms can strongly influence soil biota communities, including enchytraeids. This influence appears dependent on the ecological group of the earthworm species involved. In a seminatural mesocosm experiment, we demonstrated that earthworm species strongly differ in their effects on enchytraeid density. A visible negative impact was observed for the larger species with higher engineering activities, such as *Lumbricus rubellus*, *Aporrectodea caliginosa*, and *L. terrestris*, which represent different ecological groups of earthworms (epigeic, endogeic, and anecic, respectively). The observed impact depended not only on the species but also on the soil profile level and earthworm density. In soil communities, understanding the ecological relationships among key groups of organisms is crucial, as this knowledge can play a significant role in maintaining and modeling species biodiversity, soil structure, and ecosystem stability.

## 1. Introduction

Earthworms inhabit most terrestrial ecosystems on our planet [1,2]. Classified as engineering organisms that alter soil structure, they facilitate water infiltration and air permeation and significantly accelerate the cycling of matter and energy flow on local and global scales [3,4]. Through their activities, earthworms also affect physicochemical properties of the soil, rates of processes occurring therein, and the living conditions of other organisms [1,5]. The density and species composition of earthworm communities depend on many factors and can range from a few individuals of a single species per square meter in poor soils and unfavorable climatic conditions to several thousand individuals of different species per square meter in fertile soils rich in humus [3,6,7,8,9]. In the natural environment, communities composed of 2–6 species are the most common, but up to 15 earthworm species can be present in a single ecosystem [1,10]. This is possible because, among other things, earthworms have evolved adaptations that allow them to exploit various ecological niches in the soil, reflecting their affiliation with diverse ecological groups, namely, epigeic, endogeic, and anecic [1,11,12].

Epigeic earthworms live in litter and topsoil horizon. Their diet consists mainly of dead plant remains and animal feces colonized by decomposing microorganisms [13,14]. Their activity leads to litter fragmentation, acceleration of its decomposition by microorganisms, and a significant decrease in its volume. The consequence of this is the limitation of living space for litter-dwelling organisms and a competition of epigeic earthworms with other litter-feeders [3,15,16]. Endogeic earthworms live in the mineral soil and consume it in search of nutrients [1]. Since mineral soil is of low nutritional quality, it is ingested and egested in large quantities resulting in bioturbation, mixing of mineral and organic materials, and formation of an extensive drylosphere network used by other soil fauna to move within the soil [4,13,14]. In addition, mucus and cast excretion by endogeics makes the drylosphere an area of high microbial activity [17]. Anecic earthworms combine the features of the two former groups [10]. They spend most of their time deep in the mineral soil, which provides them shelter from unfavorable conditions and predators. They feed on plant debris, which they collect from the soil surface and transport it to their burrows, creating so-called middens around the entrances (collected leaves mixed with earthworm coprolites and mucus) functioning as centers of decomposition and microbial activity [18,19,20]. The biotopes inhabited by their colonies are often characterized by absence of fallen leaves and high heterogeneity caused by middens. In this way, anecic earthworms reduce the habitat of epigeic animals and cause them to concentrate around the middens [12]. In particular, some species of enchytraeids use middens more readily than others, causing significant changes in the structure of soil fauna community [21].

In addition to earthworms, enchytraeids are among the most numerous groups of soil fauna [22,23]. They belong to the mesofauna (body size within 0.2–2.0 mm [24]) and reach densities of several hundred to three hundred thousand individuals per square meter [25,26]. Enchytraeids are most numerous in the upper layers of acidic soils with a large amount of humus, such as forest soils [25,27]. They feed mainly on microorganisms and organic matter and can be competitors for other groups of soil detritivores [14,22,28,29]. In the detritus food web, enchytraeids constitute an important link between microorganisms and predatory invertebrates. On the one hand, they influence microbial activity, and, on the other, they provide food for higher levels of the detritus food web [30].

Earthworms can affect soil fauna both directly—by consuming or competing for food resources—and indirectly, as engineering organisms, by modifying and transforming the soil profile [6,31]. Microorganisms (i.e., protozoa, fungi, and bacteria), nematodes, and mesofauna remains have been found in the digestive tracts of various earthworm species, although no evidence of their deliberate preying on soil fauna has been confirmed thus far [1,13,32,33]. Damage to and consumption of specific fungi by earthworms may limit the food base of fungivorous mesofauna. On the other hand, some microorganisms consumed are not digested and are excreted with coprolites in concentrations exceeding their density in the surrounding environment, and their state of increased activity can persist for many weeks [34]. This may stimulate mesofauna populations, which benefit from various nutrients contained in earthworm coprolites. This mechanism may, therefore, explain the positive impact of earthworms on the density of certain mesofauna groups reported in the literature [26]. In addition to their trophic impact, earthworms influence the soil system through a range of engineering activities. Mixing soil and litter and creating drilosphere cause significant changes in the structure of the soil profile. Mucus secretion at different levels of the profile also influences the activity of microorganisms, which serve as food sources for various mesofauna groups [3,26,35,36].

The impact of earthworms on enchytraeids is crucial for the functioning of soil communities and has been the subject of many field and laboratory studies conducted in various biotopes and with various earthworm species. The results are ambiguous and sometimes even contradictory. Most results—often based on studies of mesocosms—show a reduction in enchytraeid populations in the presence of earthworms [35,37,38,39,40]. On the other hand, there are reports of both positive impacts of earthworms on enchytraeids [21,41] or no impacts [42]. There may be multiple reasons for this inconsistency, from earthworm species affiliation to different ecological groups and their density variations, to the variations in research methodology, making it difficult to compare and interpret results. Furthermore, it should be noted that interspecific interactions in earthworm communities can affect not only earthworms themselves [43,44,45] but also the functioning of soil fauna, in particular enchytraeids [39,46]. Overall, definitive conclusions on the impacts of individual earthworm species on enchytraeids are still lacking.

The present study is a part of a complex experiment investigating the effects of density variations in lumbricid species populations on the functioning of soil communities in a European fertile deciduous forest. For this purpose, we selected five common species often dominating in European earthworm communities and representing the following three ecological groups: (1) epigeic—*Dendrobaena octaedra* (Savingy 1826) and *L. rubellus* (Hoffmeister 1843); (2) endogeic—*Allolobophora chlorotica* (Savingy 1826) and *Aporrectodea caliginosa* (Savingy 1826); and (3) anecic—*L. terrestris* (L. 1758). In the previous papers, various density-dependent responses of lumbricid populations and a significant stimulation of microbial respiration in the gradient of earthworm density rise were revealed [47,48]. In the present paper, we analyze the responses of an important mesofauna group—enchytraeid communities—within the ranges between the moderate and high densities of single-species earthworm populations in the natural sites. Enchytraeid responses were examined at two soil horizons—litter and mineral soil—and at the entire mesocosm as a sum of the soil layers. We hypothesized that (1) in the presence of earthworms, the density of enchytraeids is reduced, (2) the strength of this effect is higher in the presence of epigeic than endogeic earthworm species of similar body size, (3) the reduction in enchytraeid populations by earthworms is expressed more in the litter than in the soil horizon, and (4) an increase in density of any earthworm species leads to a higher reduction in the density of enchytraeids.

## 2. Materials and Methods

### 2.1. Preparation of Substrates and Mesocosm Setup

The litter, soil, and soil organisms used in mesocosm experiments were collected in the Masurian Lake District (northeastern Poland, 53°43′ N and 21°36′ E), in a beech–oak forest with some linden. The substrates for the experiment were collected and prepared one month before the experiment. The litter, which reached a depth of 2–4 cm, was poorly stratified (L + F/H) and composed largely of oak, beech, and linden leaves. According to the guidelines of the Food and Agriculture Organization of the United Nations, the soil was classified as haplic arenosol. The depths of soil profiles A, Bv, and BvC were in the ranges of 0–15, 15–40, and 40–90 cm, respectively, and the pH (H_2_O) levels of these layers were 5.5, 5.3, and 5.8, respectively. The percentages of carbon and nitrogen for the litter were 37.3% and 1.6%, respectively, and for the soil (in layer A), 2.7% and 0.17%. For the experiment, the litter and topsoil layer from layer A (to a depth of 15 cm) were collected separately. The litter was hand-sorted to remove woody parts of trees and shrubs, young plant seedlings, and earthworms and large predators that may prey on earthworms, then thoroughly mixed, and divided into 100 g portions. The soil was sieved through a 4 mm mesh sieve. This removed plant roots, stones, and hard plant fragments, as well as earthworms and large earthworm predators. After sieving, the soil was thoroughly mixed and divided into 10 kg portions. The mesocosms were made of plastic pipes with a diameter of 20 cm and a height of 60 cm, which were secured at the bottom with a 60 µm mesh netting and at the top with a tightly fitting plastic lid, into which a hole had been cut and sealed with mesh to allow air to enter. The design of the mesocosms prevented animal migration. Before filling the mesocosms with the previously prepared substrates, they were placed in a beech and oak forest located on the grounds of the Polish Academy of Sciences in Mikołajki (Masurian Lake District, northeastern Poland, 53°78′ N and 21°58′ E). In each mesocosm, a soil profile was created from previously prepared substrates, namely, 10 kg of soil and 100 g of litter. The mesocosms were embedded into the soil profile so that the soil profile levels within them were aligned with the corresponding layers of the surrounding soil profile.

### 2.2. Soil Fauna

Earthworms used in the experiment were collected 3 weeks prior and stored in 4 L containers at 4 °C. *L. terrestris* was collected using a 0.15% formalin solution injected directly into the earthworm burrows. *Al. chlorotica*, *D. octaedra*, *L. rubellus*, and *A. caliginosa* were collected by hand-sorting the soil and litter. The microfauna and mesofauna used in the experiment were added to the mesocosms, together with the substrates. Therefore, it can be assumed that at the beginning of the experiment, the structure and density of individual faunal groups were similar to those of natural ones.

### 2.3. Experimental Conditions

A mesocosm experiment was conducted over five full months from July until November, when the average monthly temperatures were 17.9 °C (July), 16 °C, 14.2 °C, 10.5 °C, and 5.2 °C (November). The experiment began with moisture at 70% of the soil’s water capacity. Soil temperature and moisture were measured using electronic meters throughout the experiment, and water was added to the mesocosms to maintain favorable conditions for earthworms. The amount of water added corresponded to the total summer/autumn rainfall of the year preceding the experiments.

The effect of the density of single-species earthworm populations was examined. In each earthworm species, a gradient of density increase (minimum–medium–maximum treatments) was established; the actual densities depended on species size. Thus, for the smaller species (*D. octaedra* and *Al. chlorotica*) the respective densities were 5–15–25 ind. microcosm^−1^; for the medium-sized animals (*L. rubellus* and *A. caliginosa*), they were 3–9–15 ind. microcosm^−1^. In the largest species (*L. terrestris*), only the following two density levels were tested: 2 and 3 ind. microcosm^−1^. The earthworm densities used in the experiment corresponded to the moderate to ranges between moderate and high densities of these species under natural conditions (Table 1). Before the experiment, the earthworms were placed in containers with moist filter paper for 24 h to empty their digestive tracts. This allowed us to determine the mass of the earthworms and select individuals so that their biomass was comparable in each replicate. Each of the 14 experimental treatments had four replicates.

### 2.4. Enchytraeid Extraction

After the experiment, the substrates from the mesocosms were divided into the following two layers: the top layer, containing earthworm coprolites and litter residue, and the bottom layer, consisting of mineral soil. Subsamples were collected from each layer to remove enchytraeids, which were flushed out using a modified O’Connor wet funnel method. Litter and soil samples were placed separately on 1 mm plastic sieves and set in glass funnels filled with water. A 40 W light bulb was then placed over each funnel. To avoid light and rising temperatures, the enchytraeids migrated to the bottom of the funnel and slid into glass test tubes attached to the ends of the funnels. After 5 h of extraction, the test tubes containing the flushed enchytraeids were separated from the funnels and transferred to a refrigerator. The following day, the enchytraeids were counted using a stereoscopic microscope.

### 2.5. Statistical Analyses

The experiment was designed for calculations using a one-way analysis of variance. The analysis was performed after verifying that the data met the assumptions; if they did not, the nonparametric Kruskal–Wallis test was performed. Changes in enchytraeid density were analyzed separately in the litter and mineral soil layers and collectively across the entire mesocosm. Enchytraeid density was compared between the control and the density gradients of individual earthworm species. After obtaining a significant test result, a post hoc test was performed, indicating which treatments showed significant differences. In most cases, a Tukey post hoc test (HSD) was used. If this test revealed no differences, an NIR test was used. A significance level of α = 0.05 was used for the statistical analysis. All statistical analyses were performed using Statistica 12.

## 3. Results

### 3.1. Effect of Epigeic Earthworm Species

The average density of enchytraeids in the control treatment (without earthworms) was 97,128 ± 12,184 ind. m^−2^, which corresponds to the densities of enchytraeids in natural forest ecosystems [49].

**Figure 1:**
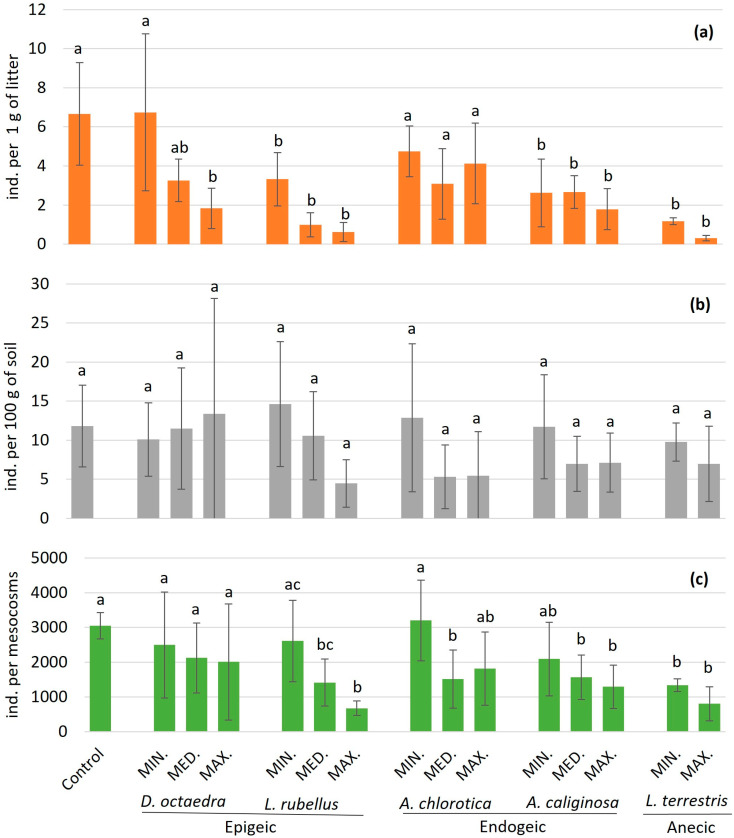
Effects of single-species earthworm populations on enchytraeid density in (**a**) 1 g of litter, (**b**) 100 g of soil, and (**c**) entire mesocosms. Min.—minimum, Med.—mean, and Max.—maximum earthworm species density. Different letters above the bars indicate significant differences compared to the control and other densities of a given earthworm species. The error bars represent ±1 SD.

*D. octaedra* significantly affected the density of enchytraeids in the litter (F_3,11_ = 4.22, *p* = 0.03). Tukey’s basic hoc tests showed significant differences between the control and the maximum earthworm density (*p* = 0.04) and between the minimum and maximum earthworm densities (*p* = 0.038). At the entire litter level, these results were confirmed (Figure 1a). At the mineral soil and entire mesocosm levels, *D. octaedra* did not cause changes in enchytraeid density (Table 2).

*L. rubellus* showed a significant negative effect on the density of enchytraeids in the litter (F_3,12_ = 13.24, *p* = 0.0004). A significant difference in the Tukey post hoc test was found when comparing the control with each of the three earthworm density treatments (Min. *p* = 0.04, Med. *p* = 0.001, and Max. *p* = 0.008; Figure 1a). The higher the *L. rubellus* density, the lower the average density of enchytraeids in the litter and the greater the statistical significance of the post hoc test comparing the treatments to the control, although there were no significant differences among the *L. rubellus* density treatments. At the mineral soil level, the effect of *L. rubellus* was not significant (Figure 1b). On the scale of the entire mesocosm, the effect of its density was significant (F_3,12_ = 9.38, *p* = 0.002; Figure 1b). The Tukey HSD post hoc test showed significant differences between the control and the medium (*p* = 0.03) and maximum (*p* = 0.003) earthworm densities. There was also a significant difference between the effect of the minimum and maximum densities of *L. rubellus* on enchytraeid abundance (*p* = 0.011).

### 3.2. Effect of Endogeic Earthworm Species

The endogeic species *Al. chlorotica* did not significantly affect the density of enchytraeids in the litter layer or in the mineral soil layer. However, a significant effect was observed on the overall abundance of enchytraeids throughout the soil profile in mesocosms (F_3,12_ = 3.54, *p* = 0.048). The NIR post hoc test showed significant differences between the control and the medium density of *Al. chlorotica* (*p* = 0.03) and between the medium and minimum density treatments (*p* = 0.02; Figure 1c).

*A. caliginosa* caused a significant decrease in enchytraeid density in the litter layer (F_3,12_ = 6.59, *p* = 0.007). The post hoc Tukey HSD test showed significant differences between the control and all analyzed *A. caliginosa* densities (Min. *p* = 0.02, Med. *p* = 0.03, and Max. *p* = 0.008; Figure 1a). The analysis did not reveal significant differences among the treatments. This species did not significantly affect enchytraeid abundance at the mineral soil level (Table 2). On the scale of the entire microcosm, *A. caliginosa* caused a significant decrease in enchytraeid density (F_3,12_ = 4.67, *p* = 0.02). The post hoc Tukey HSD test showed significant differences between the control and maximum (*p* = 0.02) earthworm densities (Figure 1c).

### 3.3. Effect of Anecic Earthworm Species

*L. terrestris* caused a significant decrease in enchytraeid density in the litter layer (F_2,9_ = 20.62, *p* = 0.0004). The post hoc Tukey HSD test showed significant differences between the control and the medium (*p* = 0.002) and maximum (*p* = 0.0007) earthworm density treatments (Figure 1a). At the mineral soil level, this species did not show a significant effect. The negative effect of *L. terrestris* on enchytraeid density was repeated across the entire mesocosm (F_2,9_ = 39.5, *p* = 0.00003). In the control, the average density of enchytraeids was approximately 3000 individuals per mesocosm; in the treatment with the minimum density of *L. terrestris*, it was 1336 individuals, and with the maximum density of this species, 808 individuals per mesocosm. Significant differences between the control and both the minimum (*p* = 0.0005) and maximum (0.0002) earthworm density treatments were noted (Figure 1c). However, no significant differences were observed among the earthworm density treatments.

## 4. Discussion

Our study revealed the negative impact of earthworms on enchytraeid density. The presence of each of the five lumbricid species caused a decrease in enchytraeid density, though the impact strength varied depending on the earthworm species, its density level, and the soil horizon. Thus, our 1st hypothesis was clearly confirmed.

A stronger negative impact was observed for the larger (*L. rubellus*, *A. caliginosa*, and *L. terrestris*) than smaller (*D. octaedra* and *Al. chlorotica*) earthworm species. However, a comparison between the impacts of epigeic and endogeic species of similar size (*D. octaedra*–*Al. chlorotica* and *L. rubellus*–*A. caliginosa*) did not show consecutive trends leaving no support for the 2nd hypothesis.

The 4th hypothesis, suggesting an increase in the negative impact on enchytraeids with earthworm density rise, was only confirmed for epigeic species, at the level of litter horizon (*D. octaedra*) or whole soil system (*L. rubellus*). In the species of other ecological groups, trends in the impact of their density gradient on enchytraeids were visible but not significant.

Regardless of the ecological group, the impact of the earthworm species was most pronounced in the litter layer with the highest abundance of enchytraeids, confirming our 3rd hypothesis. This was translated into effects at the entire mesocosm level. None of the earthworm species caused significant changes in the density of enchytraeids in the mineral soil. For epigeic and anecic species, this was likely explained by their prevailing litter/topsoil activities; for some considerations about endogeic species, see below. Table 3 summarized the data in the literature on the effects of monospecific earthworm populations on the density of enchytraeids. In general, they support our findings. However, the origin and relative significance of the mechanisms controlling these effects have been poorly studied so far, and the dominant opinion suggests that earthworm/enchytraeid relationships have a complex, often species-specific character [22,35,50]. The negative effects of earthworms on enchytraeids have sometimes been attributed to trophic mechanisms, such as competition or occasional earthworm ingestion [32,51,52]. However, Karaban and Uvarov [36], in a laboratory experiment, managed to differentiate the negative trophic effects of lumbricids (i.e., competition for resources leading to the reduction in the litter as food source and shelter) and their non-trophic activities (such as bioturbation, mucus extraction, and cast production) having, in general, a positive influence on enchytraeid populations. The balance between trophic and non-trophic impacts results in a cumulative effect of an earthworm species’ presence on enchytraeids [35].

For example, in epigeic *L. rubellus* the cumulative balance of trophic/non-trophic impacts was negative [35], which corresponds to the results of the present study. Here, the negative effect of *L. rubellus* on enchytraeid density was observed both in the litter and throughout the whole soil system (mesocosm) and progressively increased with earthworm density rise. Estimates of *L. rubellus* activities showed that by the end of the experiment it consumed up to 1/4 of the litter carbon and exerted a substantial trophic pressure on the microbial community [47,48], likely causing a strong depletion of available resources for enchytraeids. Thus, resource competition was presumably a significant factor explaining the effects of *L. rubellus* on enchytraeids. Similarly, in a lab experiment by Haimi and Boucelham [53], *L. rubellus* effectively removed birch litter from the soil surface, which was associated with a drop in Enchytraeidae numbers (Table 3). The effects of *L. rubellus* on enchytraeids on peat soils partly supported our results (Table 3); however, the results are difficult to compare due to differences in the site and experimental conditions.

The effects of the second epigeic species, *D. octaedra*, on enchytraeid density depended both on its density and the soil horizon. In the litter, at minimum, *D. octaedra* density no effect was observed, whereas at maximum density a significant decrease in enchytraeid abundance occurred; in contrast, no effect in the soil or at the level of the entire mesocosm was found. Considering the estimates of the litter consumption by this species [47,48], its effects were likely similar though weaker than in the case of *L. rubellus*: a consumption of up to 1/5 of the litter C and strong processing of the remaining litter (Max. treatment) had strongly changed the environment for enchytraeids in this horizon. Our are supported by the literature (Table 3). Thus, Hyvönen et al. [32] explained a considerable decrease in the biomass of *C. sphagnetorum* (dominant in a limed coniferous mor soil) by competition for food. As well, competitive interactions for common resources and suppression of enchytraeids by *D. octaedra* were experimentally shown by Huhta and Viberg [52] for a spruce/mixed forest in Finland.

The visible impact of the presence of endogeic *A. caliginosa* on enchytraeid density in our experiment was negative, both in the litter and in the entire mesocosm. A certain effect of *A. caliginosa* density rise was also observed. For *Al. chlorotica*, only one significant result was observed—a decrease in enchytraeid density at the level of the entire mesocosm at Med. treatment. Surprisingly, none of the endogeic species studied, even at high densities, showed a significant effect on enchytraeids in the mineral soil.

Mechanisms of the effects of endogeic lumbricids on enchytraeids are poorly understood and should be further studied. Calculations show that in both endogeic species the maximum alimentary demands during the present experiment was no more than 5% C of the amount available in the upper 10 cm of soil horizon; in addition, their moderate grazing likely supported metabolically active microbial populations [47,48]. Thus, no overgrazing of food resources by endogeics occurred, and trophic competitive relationships hardly affected enchytraeids in the mineral soil but do not explain their negative responses in the litter horizon. Moreover, in a lab experiment by Karaban and Uvarov [35], where *A. caliginosa* was deprived of access to surface litter, it exerted a rise in enchytraeid density attributed to its non-trophic activities, in particular bioturbation and mucus production. In the present study, in contrast, endogeic earthworms freely contacted with the litter horizon, which resulted in a strong enrichment of litter, with their cast material poor in available nutrients. This could sufficiently worsen trophic conditions for litter enchytraeids, which could result in their density reductions.

The literature on the relationships between endogeic earthworms and enchytraeids is nearly absent. In research conducted in orchards, Górny [37] demonstrated a negative effect of *A. caliginosa* on enchytraeid density, which supports the results of the present experiment. For *Al. chlorotica*, we found no literature data and, to our knowledge, this is the first information on the effect of *Al. chlorotica* on enchytraeids.

The effect of the anecic *L. terrestris* was strong regardless of its density. Both the Min. and Max. treatments showed reduced enchytraeid abundance in the litter and in the whole mesocosm compared to the control. A strong reduction in enchytraeid populations is easily explained by the fact that the earthworms nearly completely destroyed the litter horizon in the mesocosms, consuming up to 1/3 of the litter C amount and moving the rest of the plant remains into their burrows [47,48]. A similar result was reported by Lagerlöf and Lofs-Holmin [54] in a microcosm laboratory experiment, where a negative impact of *L. terrestris* on enchytraeids was observed, related to environmental change and reduction and transformation of the litter layer.

Taking into account a strong ‘engineering’ activity increasing site heterogeneity and specific organization of settlings in *L. terrestris*, some authors compared their effects within and at a distance of burrow middens (Table 3). Thus, Schlaghamerský et al. [21] and Nuutinen et al. [41] reported higher densities of enchytraeids in the middens compared to the adjacent non-midden areas. Middens of *L. terrestris* are functioning as biological hotspots, where the accumulated resources and favorable moisture levels support elevated microbial and animal activities (Nuutinen et al. [41]). However, a translation of a local effect to the ecosystem scale needs caution and for both studies is situation-specific. In North American hardwood forests, mean enchytraeid densities in the areas non-invaded and heavily invaded by *L. terrestris* (with a very high midden concentration) did not differ significantly (Schlaghamerský et al. [21]). In a study by Nuutinen et al. [41], an artificial introduction of *L. terrestris* to a heavy clay soil of leaching field in Finland after 17 yr caused an increase in the density of enchytraeids, which benefitted from an increased soil porosity and were nearly absent beyond the range of *L. terrestris*. A direct comparison of these studies (investigating long-term succession changes of soil communities) with our results is difficult.

In terms of ongoing invasions, only *Al. chlorotica* does not appear in the literature as an invasive species. The other species that we studied were identified as highly invasive in North America and, to some extent, in Siberia [45,55,56]. In the context of changes in earthworm communities, it is crucial to understand their environmental impact. This is particularly important for biodiversity, which undoubtedly changes when some organisms affect soil system heterogeneity and directly and indirectly negatively impact the density of others. Both earthworms and enchytraeids are soil engineers that change soil structure and affect nutrient availability for other organisms [57,58]. Enchytraeids are also described as bioindicators of chemical stress, and their activity has a significant influence on soil aeration [59]. Studies of mechanisms regulating the interactions between both groups are at the very beginning [35,60]. Understanding the mechanisms that determine the occurrence and density of key soil organism groups is important not only for natural communities but also for soil structural dynamics, which translates into soil quality.

**Table 3:** Summary of literature data on the impact of single-species earthworm populations on the density of enchytraeids. The abbreviations EP, EN, and AN denote ecological groups—epigeic, endogeic, and anecic, respectively. Arrows indicate the direction of earthworm impact on enchytraeid density: ↓ a decrease or ↑ increase in density; n.s.—no effect.

No.	Earthworm Species	Earthworm Ecological Group	Experimental or Field Data	Direction of Impact	Source
1	*Lumbricus terrestris*	AN	Seminatural experiment in mesocosms	↓	[54]
3	*Lumbricus terrestris*	AN	North American deciduous forests	↑↓	[21]
4	*Lumbricus terrestris*	AN	Agricultural fields in Finland	↑	[41]
5	*Aporrectodea caliginosa*	EN	Orchards	↓	[37]
6	*Aporrectodea caliginosa*	EN	Seminatural experiment in mesocosms in deciduous oak–beech forest	↑	[35]
7	*Lumbricus rubellus*	EP	Laboratory and field experiments on forest soils	↓	[53]
8	*Lumbricus rubellus*	EP	Peat bog experiment—different earthworm densities	↓	[61]
9	*Lumbricus rubellus*	EP	Experiment on peat meadows—different earthworm densities	n.s.	[42]
10	*Lumbricus rubellus*	EP	Seminatural experiment in mesocosms in deciduous oak–beech forest	↓	[35]
11	*Dendrobaena octaedra*	EP	Laboratory experiment on soil from a coniferous forest	↓	[32]
12	*Dendrobaena octaedra*	EP	Seminatural experiment in mesocosms, coniferous spruce forest	↓	[52]
13	*Dendrobaena octaedra*	EP	Seminatural experiment in mesocosms on soil from spruce and pine forests with the addition of pine and birch litter	↓	[62]

## 5. Conclusions

Our research shows the negative effects of five globally important earthworm species on enchytraeid density in the soils of a Central European deciduous forest. The selected earthworm species represent three ecological groups. Four of them (*D. octaedra, L. rubellus, L. terrestris,* and *A. caliginosa*) are described in the literature as invasive species that significantly impact the functioning of soil ecosystems. The strength of earthworm impact varies and depends on (1) the earthworm species, (2) its ecological group, (3) earthworm density, and (4) horizon of the soil profile. A decrease in enchytraeid density in the presence of earthworms suggests direct and indirect mechanisms of earthworm effects on enchytraeids. Significant results were obtained for the litter horizon and for the entire soil profile created in mesocosms. None of the earthworm species caused changes in enchytraeid density in the mineral soil. For four lumbricid species (*D. octaedra*, *L. rubellus*, *A. chlorotica*, and *A. caliginosa*), we demonstrate that enchytraeid populations negatively responded (or strongly tended to respond) to earthworm density rise either in the litter or at the level of the whole mesocosm. For the first time, information on the effects of *Al. chlorotica* on enchytraeid density was revealed.

Our results allow for a better understanding of the ecological relationships occurring in the soil system. Furthermore, they enable predictions of changes in enchytraeid density when earthworms enter the ecosystem. The data can also be used to develop and calibrate models describing the relationships among key groups of soil fauna.

## Figures and Tables

**Table 1:** Experimental setup. Gradients of earthworm species density (expressed per mesocosm, MK, or per m^2^), from the Control treatment (no earthworms) to Maximum treatment, - no treatment.

Ecological Group	Earthworm Species		Earthworm Density					
		Control	Minimum		Medium		Maximum	
			MK	m^2^	MK	m^2^	MK	m^2^
Epigeic	*Dendrobaena octaedra*	0	5	155	15	465	25	775
Epigeic	*Lumbricus rubellus*	0	3	96	9	287	15	478
Endogeic	*Allobophora chlorotica*	0	5	155	15	465	25	775
Endogeic	*Aporrectodea caliginosa*	0	3	96	9	287	15	478
Anecic	*Lumbricus terrestris*	0	2	64	-		3	96

**Table 2:** Effects of earthworms on enchytraeid density. One arrow shows that in the presence of earthworms enchytraeid density was reduced compared to the control. The 2nd arrow shows that the negative effect was aggravated with density rise of a given species; n.s.—no significant effect.

Earthworm Species	Earthworm Ecological Group	Enchytraeid Density		
		Litter Level	Soil Level	Mesocosms (Litter + Soil)
*Lumbricus terrestris*	Anecic	↓	n.s.	↓
*Aporrectodea caliginosa*	Endogeic	↓	n.s.	↓↓
*Allobophora chlorotica*	Endogeic	n.s.	n.s.	↓↓
*Lumbricus rubellus*	Epigeic	↓	n.s.	↓↓
*Dendrobaena octaedra*	Epigeic	↓↓	n.s.	n.s.

## Data Availability

The data presented in this study are available on the internal servers of Cardinal Stefan Wyszynski University. The corresponding author will make the raw data available to all interested parties who contact him by email.
